# Design and Optimization of 3D-Printed Tablets Containing Mucuna Extracts for Erectile Dysfunction Management: A DoE-Guided Study

**DOI:** 10.3390/plants13162294

**Published:** 2024-08-18

**Authors:** Ratchapoom Wattanawiggan, Sunee Chansakaow, Pensak Jantrawut, Pattaraporn Panraksa, Jutamas Jiaranaikulwanitch, Suruk Udomsom, Patnarin Worajittiphon, Pratchaya Tipduangta

**Affiliations:** 1 Department of Pharmaceutical Sciences, Faculty of Pharmacy, Chiang Mai University, Chiang Mai 50200, Thailand; ratchapoom_wat@cmu.ac.th (R.W.); sunee.c@cmu.ac.th (S.C.); pensak.j@cmu.ac.th (P.J.); pattaraporn.pan@cmu.ac.th (P.P.); jutamas.jia@cmu.ac.th (J.J.); 2 Biomedical Engineering Institute, Chiang Mai University, Chiang Mai 50200, Thailand; suruk.u@cmu.ac.th; 3 Department of Chemistry, Faculty of Science, Chiang Mai University, Chiang Mai 50200, Thailand; patnarin.w@cmu.ac.th

**Keywords:** 3D printing, erectile dysfunction, DoE, Mucuna extract, optimization, sustained release

## Abstract

Erectile dysfunction (ED) refers to the inability of the penis to maintain a firm erection during sexual activity. Mucuna, or *M. pruriens*, contains levodopa, a compound showing promise in ED treatment. However, formulating Mucuna extract into tablet dosage forms is challenging due to its semisolid nature. This study aimed to develop sustained-release tablets containing Mucuna extract via semisolid extrusion 3D printing. Eudragit RS PO (Eudragit) served as a sustained-release polymer, with poly (vinyl alcohol) (PVA) as a co-polymer for forming the tablet matrices. This study had the following two main phases: screening, which identified the factors affecting the printability, and optimization, which focused on the factors influencing the levodopa release and its consistency. The results showed that both the polymeric solid percentage content (PSPC) in the semisolid slurry and the Eudragit-PVA ratio significantly affected the printability. All of the formulations were printable, and the PSPC and Eudragit-PVA ratios were incorporated into the optimized model. The desired formulation, achieving targeted levodopa release and consistency, had a PSPC of 58.8% and a Eudragit-PVA ratio of 2.87:1. In conclusion, semisolid extrusion 3D printing guided by the design of experiments (DoE) proved feasible for producing reliable 3D-printed tablets with consistent active ingredients and desired release rates.

## 1. Introduction

Erectile dysfunction (ED) denotes a medical condition characterized by the inability of males to attain a complete erection during sexual activity. An estimated 150 million men are affected by ED, with a projected increase to 322 million by the year 2025 [1]. The mechanism of achieving an erection is intricate, involving neural signals triggering the dilation of blood vessels in the penile region, facilitating blood influx and subsequent erection. Disruptions in this intricate process can culminate in ED, stemming from various physical or psychological triggers [2]. Treatment usually begins with lifestyle adjustments and may include phosphodiesterase 5 (PDE5) inhibitors such as sildenafil, vardenafil, tadalafil, and avanafil. These medications assist in achieving erections by inhibiting the enzyme PDE5 and boosting nitric oxide (NO) production [3,4].

There is an increasing interest in the utilization of natural substances for the management of ED. Various substances such as *Panax ginseng*, Pycnogenol, Prelox, and *Tribulus terrestris* have exhibited promising potential in augmenting the synthesis of NO [5]. Studies have indicated that extracts from *Mucuna pruriens* seeds could enhance male sexual potency in aged mice [6], and might offer therapeutic benefits for ED in individuals by elevating NO production and regulating cyclic guanosine monophosphate (cGMP) levels [7,8]. Notably, the presence of levodopa in the extract enhances sexual responses by acting on dopamine receptors in the brain [9,10]. Since Mucuna extract includes various compounds that contribute to treating ED, in which levodopa is present as one of its major components, it was employed as a marker to measure the effectiveness of the treatment for ED [11]. Existing research has demonstrated the safety of Mucuna extracts in animal studies, and the use of sustained-release formulations could potentially enhance the management of medications and the quality of life for patients [12,13].

Presently, there is a study on controlled-release Viagra that can minimize the risk of side effects, make the drug more accessible for men with pre-existing conditions, supporting their psyche, and enhance their overall quality of life [13]. In addition, levodopa, which plays an important role in treating ED by using Mucuna extract, has a short plasma half-life (approximately 90 min) [14]. Thus, the development of sustained-release formulations from Mucuna extracts to alleviate ED symptoms aims to promote convenience in medication management for ED patients and improve their quality of life. For example, patients with ED would not need to plan their sexual activities in advance because sustained-release formulations can prolong their effects over a longer period compared to conventional formulations.

The goal of sustained-release drug delivery systems is to maintain steady drug levels in the plasma, reduce the dosing frequency, improve compliance, minimize side effects, and maximize the treatment effectiveness [15]. Sustained-release Mucuna extract formulations will offer a natural approach to treating ED, allowing patients to engage in sexual activity without planning. Developing sustained release formulations requires selecting suitable materials. Eudragit^®^ is a series of synthetic polymers utilized in pharmaceutical formulations. Eudragit^®^ is biologically inert, non-absorbable, and non-toxic. Different types of Eudragit^®^ are available, each designed to control drug release in various formulations [16]. In this study, Eudragit RS PO (Eudragit) was used as a polymer for sustained release, and polyvinyl alcohol (PVA) was used as a co-polymer to maintain the shape of the tablets. Three-dimensional printing (3DP) technology is well-suited for creating such a sustainable dosage form. The semisolid extrusion (SSE) 3DP technique uses gel or paste materials to create controlled-release tablets [17]. This technique is particularly suitable for the viscous liquid form of Mucuna extract, making it ideal for producing sustained-release tablets. Due to the various parameters involved in 3DP, the design of experiments (DoE) approach was employed to aid in screening and optimizing the formulation of these tablets. This study aimed to develop sustained-release tablets containing Mucuna extract using a semisolid extrusion 3D printer.

## 2. Results and Discussion

### 2.1. Screening Factors Affecting Polymer Matrix Formulation Containing Mucuna Extract in 3D Printing

The preliminary study aimed to determine the optimal printing parameters. The results showed that Eudragit, as a single polymer, was not suitable for the 3D printing of the tablets. However, polymer blends provided a solution. A blend of polymeric solid percentage content (PSPC) in the semisolid slurry at 50% and a Eudragit-PVA polymer ratio (PR) at 50% proved to be printable. This highlights the complexity of 3D printing factors, making it crucial to screen these factors to understand their impact on 3D printing. Supplementary Table S1 shows a comprehensive overview of the screening formulations utilized in the study, encompassing several critical factors essential for the formulation process. These factors included the PSPC, PR, glycerin percentage, and isopropyl alcohol (IPA) percentage. The printed tablets are hemispherical, black, and slightly flexible, as shown in Supplementary Figure S1. All of the formulations underwent successful printing processes, resulting in tablet shapes with a distinct hemispherical characteristic.

**Figure 1:**
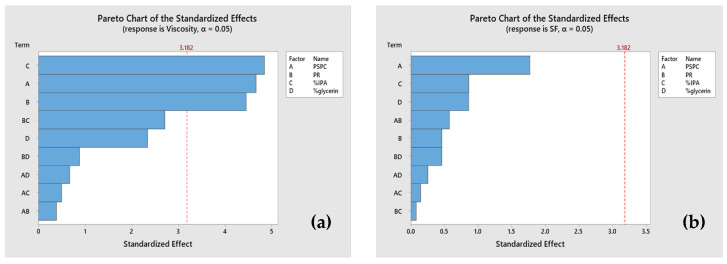
Pareto charts showing the effect of (**a**) factors on the viscosity and (**b**) shape fidelity.

Viscosity and shape fidelity right after printing were used as the outcome to screen the 3D printing parameters, since a lower viscosity, falling below the minimum viscosity requirement of 30 Pas for the printing ink used by the extrusion printer [18], can affect the quality of the printed structure. Shape fidelity is important because it ensures that the printed parts meet the design specifications, function correctly, and maintain their structural integrity. A shape fidelity that is close to one shows that the printed tablets accurately match their intended design [19]. Figure 1a shows that the concentrations of the PSPC, PR, and % IPA significantly impact the viscosity (*p*-value < 0.05). Specifically, higher PSPC concentrations correlate with increased viscosity, while a higher Eudragit content, indicated by the PR, leads to a decreased viscosity. Moreover, there is an inverse relationship observed between the viscosity and % IPA, with viscosity decreasing as the percentage of IPA increases. The shape fidelity results indicate that most of the printed formulations approached a shape fidelity value close to one, primarily influenced by the viscosity. Notably, lower viscosity levels are associated with a reduced shape fidelity during the 3D printing process [18], emphasizing the intricate relationship between the viscosity and printing accuracy. This highlights the importance of depositing a stable shape of the semisolid filament precisely in the desired location during extrusion. However, despite the significant influence of the viscosity on the shape fidelity, none of the aforementioned factors were found to have a significant impact on the fidelity of the tablet shapes, as shown in Figure 1b. This result shows that the range of factors in the screening formulation can be used to fabricate 3D tablets with high accuracy, as the shape fidelity of most formulations is close to one.

The differences between the shape fidelity results are presented in Supplementary Table S1 and the images shown in Figure S1 are attributed to the stages of the tablet’s solidification process. The images in Figure S1 depict the tablets after complete solidification, where structural collapses may occur due to solvent evaporation. This solidification process increases the rigidity of the printing inks and the 3D-printed tablets, which could cause the tablets to collapse under their own weight [20,21]. Conversely, the shape fidelity measurements presented in Supplementary Table S1 are based on images taken immediately after the printing process (Supplementary Figure S2), when the tablets were still in a semisolid state and had not yet undergone significant evaporation or structural collapse.

### 2.2. Levodopa Quantitation

**Figure 2:**
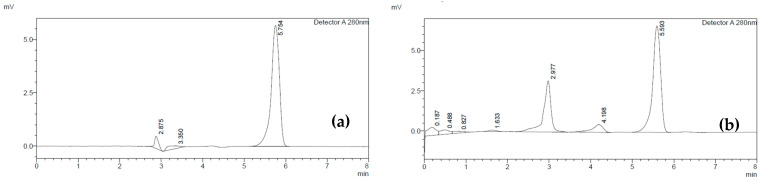
Chromatographic results of the specificity samples: (**a**) standard levodopa; (**b**) levodopa spiked in placebo.

The main goal of this study was to develop a robust and dependable Reversed-Phase High-Performance Liquid Chromatography (RP-HPLC) method specifically designed for accurately quantifying levodopa in the Mucuna extract tablets. The analysis demonstrated a levodopa peak in the sample could be separate from the other ingredients, as depicted in Figure 2, and a strong linear correlation between the concentrations of levodopa and the corresponding peak areas, with a coefficient of determination (r^2^) of 0.9964. The percentage recovery was 102.36% and the % RSD of the inter-day and intra-day precision were 0.50 and 0.44.

### 2.3. Formulation Optimization and Release Study

In this phase of the study, attention was directed towards optimizing the crucial parameters for the formulation of sustained-release tablets containing Mucuna extract. After careful consideration, the selected factors for the optimization were identified as the PSPC and PR due to their substantial influence on the viscosity, a critical determinant of tablet manufacturability and performance. Conversely, the percentage of IPA was excluded from the optimization process. Despite its potential influence on the viscosity, the percentage of IPA was found to accelerate the solidification process of the tablets without perceptibly affecting the SF, thus indicating its negligible impact on the printability. The primary objective of the current phase was to refine the formulation parameters to attain desired sustained-release characteristics for the Mucuna extract tablets. The optimization formulation (F) components, including Mucuna, the PSPC, PR, glycerin, and % IPA, which were created by the DoE process, are reported in Supplementary Table S2. These formulations encompassed variations in the PSPC and PR concentrations to investigate their synergistic effects on the tablet performance.

**Figure 3:**
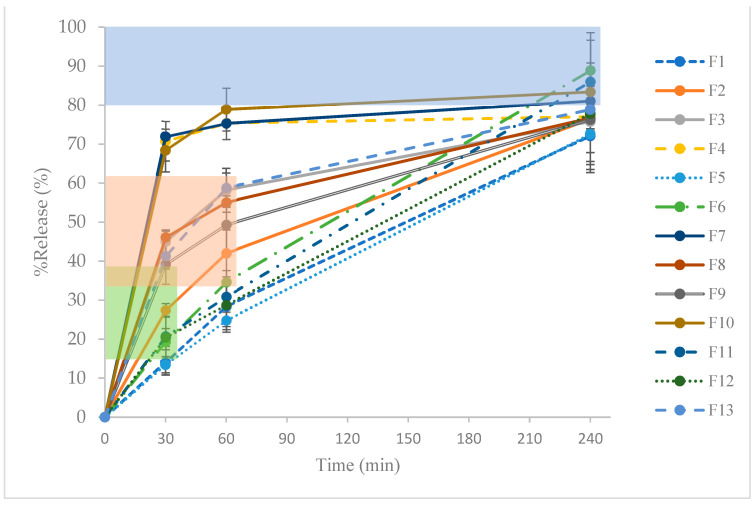
Released profiles of formulations 1–13. The green area shows the specific criteria of the % release at 30 min (14–39%), the orange area shows the specific criteria of the % release at 60 min (36–61%), and the blue area shows the specific criteria of the % release at 240 min (not less than 80%) according to the USP Monograph of Carbidopa and Levodopa Extended-Release Tablets.

In order to assess the levodopa sustained-release properties from the 3D-printed Mucuna extract tablet, we used a release criterion from the United States Pharmacopeia (USP) Monograph of Carbidopa and Levodopa Extended-Release Tablets, which is a worldwide accepted standard. The rationale of using this monograph is because it has a similar drug (levodopa) and drug-release period of up to 6 h, which mimics our purpose for the sustained release of the Mucuna extract for treating ED. The assessment of the release profiles shows promising results, with specific formulations meeting predefined criteria according to the USP Monograph of Carbidopa and Levodopa Extended-Release Tablets at different time intervals. Figure 3 illustrates the levodopa release from the 13 formulae of the 3D-printed tablets containing the Mucuna extract. The acceptable levodopa ranges are highlighted in green (14–39%), orange (36–61%), and blue (NLT80%) for 30, 60, and 240 min. Notably, at 30 min, formulations F1, F2, F6, F11, and F12 exhibited percentages of release values that complied with the specified criteria. Similarly, at 60 min, formulations F2, F3, F8, F9, and F13 demonstrated release profiles meeting the desired benchmarks. Furthermore, at the 240 min mark, formulations F6, F7, F10, and F11 released levodopa above 80%. The average content of levodopa was reported to be between 61.64% and 85.57%. This variation in the levodopa content was associated with the PSPC and PR, as both parameters can influence the viscosity of the semisolid slurry. Our findings support the observations of Kumar et al. [22], who suggested that formulations with a higher viscosity may exhibit a decrease in drug content. Eight formulations (F1, F2, F5, F6, F9, F11, F12, and F13) displayed a % RSD exceeding 7.3%.

**Figure 4:**
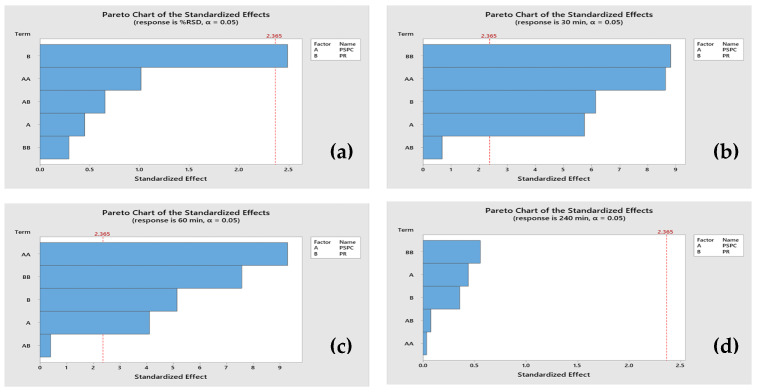
Pareto charts showing the effect of the PSPC (A) and PR (B) on the % RSD (**a**), % release at 30 min (**b**), % release at 60 min (**c**), and % release at 240 min (**d**).

The obtained experimental data of the % RSD of the levodopa content and the percentage of the levodopa release at 30 min, 60 min, and 240 min were analyzed to draw a relationship between both the PSPC and PR, as shown in Figure 4. It was observed that the PR was a significant factor impacting the % RSD of the formulations (*p*-value < 0.05). Additionally, both of the percentages of the levodopa release at 30 min and 60 min showed significant impacts to both the PSPC and PR, and their squared values. However, it is important to note that the % release at 240 min was statistically insignificantly associated with the examined factors.

The correlation between the PSPC and PR and the percentage of the levodopa release at 30 and 60 min fit with Quadratic model. Equations (1) and (2) show the relationship of the PSPC and PR to the percentage of the levodopa release at 30 and 60 min. Specifically, higher levels of both correlated with a decreased percentage of levodopa release at 30 and 60 min.

30 min = 767.4 − 20.48 PSPC − 4.027 PR + 0.1664 PSPC × PSPC + 0.04253 PR × PR − 0.0087 PSPC × PR(1)

60 min = 792.5 − 21.61 PSPC − 3.555 PR + 0.1765 PSPC × PSPC + 0.03596 PR × PR − 0.0051 PSPC × PR(2)

**Figure 5:**
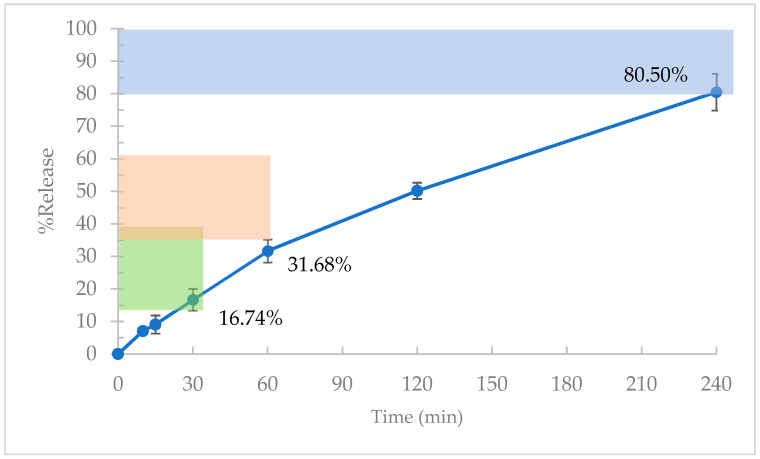
Release profiles of the optimized 3D-printed tablets containing Mucuna extract formulae. The green area shows the specific criteria of the % release at 30 min (14–39%), the orange area shows the specific criteria of the % release at 60 min (36–61%), and the blue area shows the specific criteria of the % release at 240 min (not less than 80%) according to USP Monograph of Carbidopa and Levodopa Extended-Release Tablets.

The experimental data were used to establish predictive modeling for optimized formulations. These formulations represent a strategic combination of the PSPC and PR aimed at achieving drug release profiles according to the USP Monograph of Carbidopa and Levodopa Extended-Release Tablets, with the % RSD values within 4.7%. The details of this optimal formulation and the corresponding results indicate the levels of PSPC at 58.8% and the PR at 2.87:1 for formulating sustained-release tablets containing Mucuna extract. The results showed that, at 30 min, the percentage of levodopa release complied with the USP monograph criteria (14–39%). However, at 60 min, it slightly failed to meet the lower limit of the levodopa release criteria (36–65%). At 240 min, the optimized formulation met the criteria of the NLT 80% release (Figure 5).

The Akaike Information Criterion (AIC) value of fitting models is 29.04 in the zero-order model, 5.784 in first-order model, 7.36 in the Korsmeyer–Peppas model, 76.81 in the Higuchi model, and −28.22 in the Hixon–Crowell model, respectively, as shown in Table 1.

The release profiles in Figure 5 show an almost linear release of levodopa in the optimized formulation, reflecting the sustained release behavior of the levodopa from the 3D-printed tablets. To describe the kinetics of the drug release from the printed tablets, the release data were analyzed according to different kinetic equations. The Hixson–Crowell model was the most suitable for describing the release of levodopa from the 3D-printed tablets. This model assumes that the drug particles dissolve uniformly, and that the surface area of the particles decreases as the drug dissolves. The dissolution process involves erosion, as fitted with the Hixson–Crowell model [23].

Despite the optimized formulation not fully meeting the USP Monograph of Carbidopa and Levodopa Extended-Release Tablets, this information is sufficient to demonstrate the sustained release concept of the 3D-printed tablets containing Mucuna extract. The optimized formula was feasible for sustaining the release of the Mucuna extract over a 6 h period.

### 2.4. Excipients and Mucuna Extract Compatibility Study

**Figure 6:**
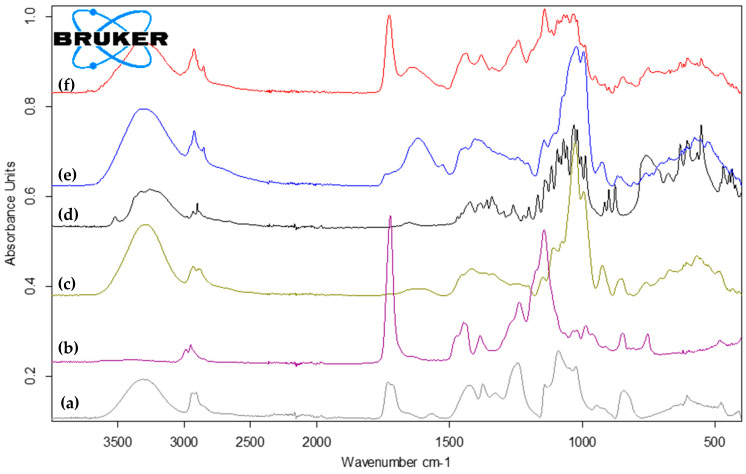
FTIR spectra of PVA (a), Eudragit (b), glycerin (c), lactose (d), Mucuna extract (e), and the 3DP tablet (f).

Fourier transform infrared spectroscopy (FTIR) analysis was conducted to explore the functional groups and interactions between the components. The molecular structures of the Mucuna extract, Mucuna tablet, and other ingredients are depicted in Figure 6. The main characteristic absorption bands of the PVA include O-H stretching at 3313 cm^−1^, the asymmetric stretching of CH_2_ at 2939 and 2911 cm^−1^, the shoulder stretching of C-O (the crystalline sequence of PVA) at 1143 cm^−1^, and the stretching of C = O and bending of O-H (the amorphous sequence of PVA) at 1089 cm^−1^, as shown in Figure 6a [24]. Figure 6b shows the FTIR of the Eudragit RS PO, including small CH_2_ stretching at 2987 and 2951 cm^−1^, a strong C = O ester stretching at 1723 cm^−1^, and characteristic bands of the ester groups at 1140–1150 cm^−1^ and 1236 cm^−1^ [25]. Glycerin exhibits its characteristic peaks, including the OH stretching broad band at 3284 cm^−1^, asymmetric CH_2_ stretching at 2933 cm^−1^, and C-O bending at 1026 cm^−1^, as shown in Figure 6c. Lactose displays characteristic bands at 2900–3250 cm^−1^, corresponding to CH_2_ asymmetric stretching, bands at 1140–1030 cm^−1^, corresponding to the intermolecular stretching of carbohydrates, and bands at 800–1000 cm^−1^, corresponding to carbohydrates, as shown in Figure 6d [26]. The Mucuna extract exhibits characteristic peaks, including O-H stretching at 3305 cm^−1^, phenyl group C = C vibrations at 1378, 1020, 995, 924, and 520 cm^−1^, and C-H at 2923 cm^−1^, as shown in Figure 6e. This finding is consistent with the reported literature [27]. Figure 6f shows the spectra of a 3D-printed tablet containing Mucuna extract and other ingredients. The characteristic peaks of the excipients are observed at their original position or with a slight shift in wavenumbers. This indicates the compatibility of the Mucuna extract and the other components. However, the FTIR served as a screening test, and further product stability testing is required to ensure the Mucuna content in the 3D-printed tablet throughout its shelf-life.

## 3. Materials and Methods

### 3.1. Materials

The Mucuna seeds were purchased from Chiang Mai province, Thailand. Eudragit RS PO was kindly gifted from EVONIK (Essen, Germany). Poly (vinyl alcohol) (PVA) was purchased from Sigma-Aldrich (St. Louis, MO, USA), with the weight-average molecular weight of 13,000–23,000 g/mol. Methanol (HPLC grade) and isopropyl alcohol (IPA, analytical grade) were purchased from RCI Labscan Ltd. (Bangkok, Thailand).

### 3.2. Plant Extraction and Preparation

Mucuna extract was supplied from the Medicinal Plant Innovation Center, Faculty of Pharmacy, Chiang Mai University, Thailand. Briefly, Mucuna seeds were washed and dried at 55 °C in a hot-air oven, followed by being roasted in a wok and ground using an electrical blender. The seed powder was extracted using a Soxhlet apparatus with 80% methanol as a solvent. Crude extracts were filtered through a Whatman No. 1 paper filter and then evaporated by a rotary evaporator (EYELA; Tokyo, Japan).

### 3.3. Screening

The screening formulations (S) were designed by the Minitab 19 (Minitab, LLC; State College, PA, USA), with a definitive screening design that is shown in Supplementary Table S1. The factors used in this design were the PSPC, the PR, the glycerin percentage as a plasticizer, and the IPA percentage as a solvent. The outcomes were the viscosity and shape fidelity (SF).

#### 3.3.1. Sustained-Release Tablet 3D Printing

**Figure 7:**
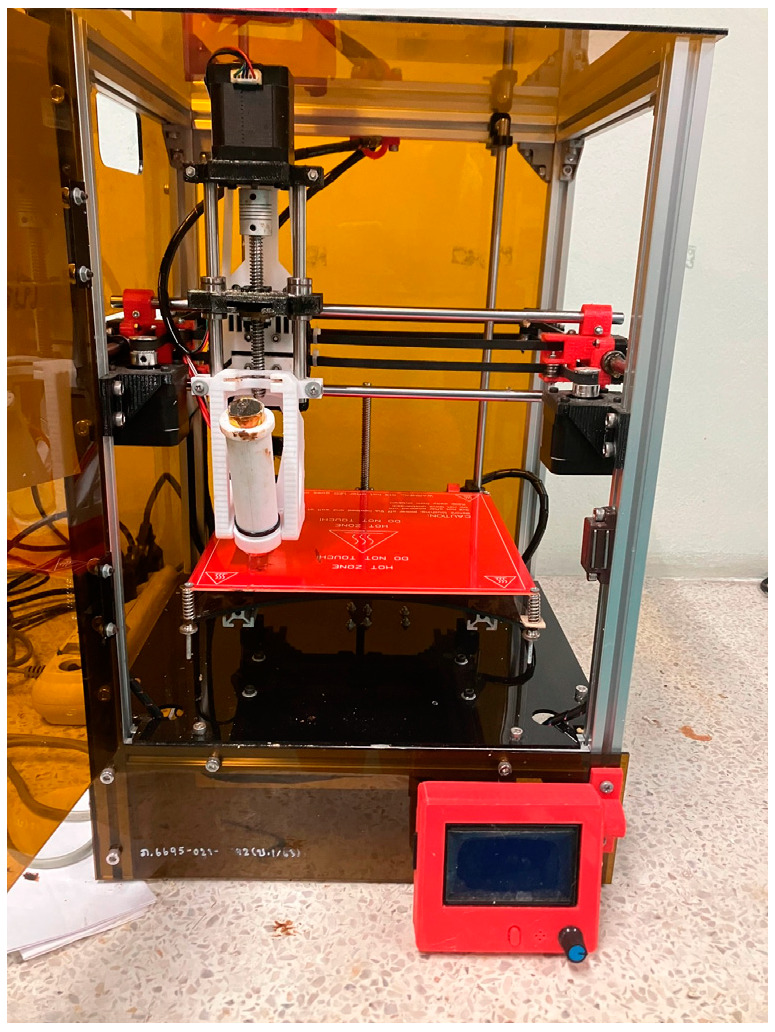
The custom-made semisolid extrusion 3D printer which was used in this study.

The semisolid extrusion 3D printer, designed and built from the Biomedical Engineering Institute, Chiang Mai University, Thailand, was used as a printer for printing the tablet shape dosage form, as shown in Figure 7. This customized syringe extrusion 3D printer is based on a core-XY 3D printer, in which an extrusion nozzle can move in an X- and Y-axis, and a separate building plate on a *Z*-axis, while printing layer-by-layer for generating a 3D structure. The syringe-based extruder used a stepper motor to move a plunger of a 10 mL syringe via a direct lead screw drive. The syringe extrusion 3D printer was controlled by a computer and a user interface on the printer. The Mucuna extract was dissolved in IPA and mixed with polymers and other ingredients via a magnetic stirrer and degassed via a sonicator. The tablets were designed by AUTODESK Tinkercad web software with a 10 × 20 × 5 mm caplet shape. The blunt-end needle 16G injector (the outer diameter was 1.63 mm) was used in this process. The syringe temperature was set at 40 °C, the base temperature was set at room temperature (25 °C), the printing speed was set at 10 mm/s, and the nozzle traveling speed was set at 120 mm/s. The 20 × 20 cm^2^ glasses were used as a base for supporting the tablets in the printing process. The tablets were dried in a hot-air oven at 40 °C for 2 h, then kept in a zipped bag that was stored in desiccator at room temperature before further use.

#### 3.3.2. Viscosity

The viscosity of each formulation was investigated using the Brookfield R/S rheometer (AMETEK Brookfield; Middleborough, MA, USA). The measurements were carried out by measuring the viscosity while varying the shear rate, ranging from 0 to 100 s^−1^ at 25 °C. The gap between the plate and base was set at 1 mm. All of the experiments were carried out in triplicate.

#### 3.3.3. Shape Fidelity

The printed tablet images were captured immediately after printing by an iPhone SE2 camera, as shown in Figure S2. Then, their areas were analyzed using ImageJ software (https://www.tinkercad.com/, accessed on 25 July 2024) (LOCI; University of Wisconsin, Madison, WI, USA). The shape fidelity factor was calculated following the protocol of Panraksa et al. [28], using Equation (3), shown below. All of the experiments were conducted in triplicate.

Shape fidelity factor = Measured area (cm^2^)/Calculated area (cm^2^) (3)

### 3.4. Chromatographic Conditions

The concentration of the levodopa was evaluated following the protocol of Dhanani et al., 2015, with minor adjustments [29]. The levodopa was analyzed using a Shimadzu LC-2030C 3D (Kyoto, Japan) RP-HPLC (Reverse-Phase High-Performance Liquid Chromatography) system. Data were acquired using software from Shimadzu Lab Solutions. Chromatographic separation was accomplished using an Ascentis C18 column (250 × 4.6 mm, i.d., 5 μm). The mobile phase was a mixture of 0.1 N formic acid and methanol (98:2). The solvent flow rate was 1.0 mL/min. The injection volume was 5 μL. The photo diode array detector wavelength was set at 280 nm. All of the experiments were conducted in triplicate.

### 3.5. Analytical Method Validation for Levodopa Determination

The analytical method for the quantification of the levodopa in the Mucuna extract tablets was validated following the ICH Q2 (R2) guideline (ICH guideline, 2023). The analytical procedure performance characteristics, consisting of the specificity, linearity, accuracy, and precision, were evaluated.

#### 3.5.1. Specificity

The specificity was evaluated by comparing the chromatograms of standard levodopa and levodopa spiked in placebo. The acceptance criteria included levodopa peaks in the sample which were able to be separated from the other ingredients.

#### 3.5.2. Linearity

The linear relationship was studied at concentrations between 1 and 20 µg/mL. Three identical injections of each analyte were made at 5 different known concentrations. Calibration curves were created using the peak area as the dependent variable, with the concentrations of the standards acting as the independent variable. The acceptance criteria included an r^2^ of not less than 0.99.

#### 3.5.3. Accuracy

The accuracy of the analytical method was assessed over its specified range at 60–150% of the test concentration using a recovery study. The accuracy of the samples included levodopa standards at three different concentration levels, 6.0, 10.0, and 15.0 µg/mL. The recovery was determined in triplicate for each concentration, and the recovery (%), which should be in the range of 80–110% [30], was calculated according to Equation (4), as follows:

%recovery = [concentration found/concentration added] × 100%(4)

#### 3.5.4. Precision

Several intra- and inter-day administrations of standard solutions of levodopa were evaluated at 100% test concentration levels to determine the precision of the method. The suggested approach was used to analyze six standard injections (10.0 µg/mL) within a single day to evaluate the intra-day precision and two consecutive days to determine the inter-day precision. The precision of this procedure was measured using the relative standard deviation (% RSD) of the levodopa content. The % RSD was not more than 7.3 [30].

### 3.6. Optimization

In the optimization study, the formulations were designed by the Minitab 19 software19 (Minitab, LLC; State College, PA, USA) with a center composite design, as shown in Supplementary Table S2. The factors used in this design were the selected factors from the screening phase. The outcomes included the percentage of the levodopa release and the % RSD of the levodopa content.

#### 3.6.1. Levodopa Release Study and Drug Release Mechanism Evaluation

A Hanson Research SR8-Plus Dissolution Test Station (Chatsworth, CA, USA) was used in the release study using apparatus 2 with 50 rpm. The medium was 0.1 N of hydrochloric acid (RCI Labscan Ltd., Bangkok, Thailand). The collecting times followed the Carbidopa and Levodopa Extended-Release Tablets USP monograph. An amount of 3 mL of the medium was withdrawn at 0.5, 1, and 4 h, and 3 mL of the medium was added to a vessel. All of the experiments were conducted in triplicate. In the optimized formulae, the levodopa content was determined at intervals of 0, 10, 15, 30, 60, 120, and 240 min. The release data were fitted with the kinetic drug release models to evaluate the mechanism of the drug release.

#### 3.6.2. Levodopa Content in Mucuna Extract Tablets

The tablets were dissolved with 50% methanol in a volumetric flask (100 mL). Then, 1 mL of the solution was pipetted into a volumetric flask (10 mL). The concentration of the levodopa was evaluated following the protocol in Section 2.4. All of the experiments were conducted in triplicate.

### 3.7. ATR-FTIR

The infrared spectra in this study were acquired by a BrukerIF-66 spectrometer (Bruker Optics, Coventry, UK) equipped with a Golden gate MKII accessory from Specac Ltd. (Orpington, UK). A few milligrams of the raw materials of a piece of printed tablet were placed on the crystal of the ATR cell; each spectrum was acquired with a data resolution of 2 cm^−1^, with 32 scans over a range of 4000 to 550 cm^−1^ at an ambient temperature.

## 4. Conclusions

In summary, the screening phase reveals that the PSPC, PR, and % IPA significantly influence the viscosity, but none significantly affect the shape fidelity. However, the linearity, accuracy, and precision of the levodopa determination all meet the Association of Official Agricultural Chemists (AOAC) criteria. In the optimization phase, it was evident that the PR notably impacted the % RSD of the Mucuna extract content as determined by the levodopa levels. Both the PSPC and PR significantly affected the percentage of the Mucuna extract released at 30 and 60 min. However, the % release at 240 min did not exhibit any statistically significant factors. The optimal formulation demonstrates promising potential for the fabrication of sustained-release tablets containing Mucuna extract as guided by the DoE for tunable release rates. Moreover, the Mucuna extract proved to be compatible with the other ingredients in the 3D-printed formulation. Our study proves the feasibility of using semisolid extrusion 3D-printed tablets containing Mucuna extract guided the DoE to produce consistent active ingredients and desirable release rates for treating ED. This study should be further investigated in terms of treating ED, and the following are the proposed directions for future development: an assessment of each dimension (e.g., the thickness, diameter, width, and length) of the 3D-printed tablets; exploring alternative agents via polymer blend studies, as well as the characteristics of blended polymers so to provide more options in polymer selection, such as the mixing of Eudragit RS PO with natural hydrophobic polymers like gum copal and gum damar; the dosage forms; optimizing the release profiles; conducting clinical trials to confirm the efficacy and safety for treating ED. However, additional inquiries are needed to assess the practical usage and provide confidence in preparing suitable dosage forms for individual patients.

## Figures and Tables

**Table 1:** In vitro kinetic of levodopa release from the optimized 3D-printed tablets.

Formulation	Zero-Order		First-Order		Korsmeyer–Peppas			Higuchi		Hixon–Crowell	
	AIC	R^2^	AIC	R^2^	AIC	R^2^	n	AIC	R^2^	AIC	R^2^
O1	29.04	0.9757	5.78	0.9945	7.36	0.998937	0.727987	76.81	0.9728	−28.22	0.9987

## Data Availability

The original contributions presented in the study are included in the article/Supplementary Materials, further inquiries can be directed to the corresponding author/s.

## Supplementary Materials

The following supporting information can be downloaded at: https://www.mdpi.com/article/10.3390/plants13162294/s1, Figure S1: Images of 13 screening formulation tablets after solidification; Figure S2: Images of 13 screening formulation tablets after printing for calculating shape fidelity; Figure S3: Coded coefficients of factors on (a) viscosity and (b) shape fidelity; Table S1: Viscosity and shape fidelity of the screening formulations; Table S2: Optimized formulations design by design of experiment.
