# Supplementary material for: Design and Optimization of 3D-Printed Tablets Containing Mucuna Extracts for Erectile Dysfunction Management: A DoE-Guided Study
Source: Plants (Basel). 2024 Aug 18;13(16):2294. doi: 10.3390/plants13162294 (PMC11360075; doi:10.3390/plants13162294)
Supplement: Supplementary file 1 [file plants-13-02294-s001.zip › plants-3151937-supplementary.pdf]

Supplementary Materials

Design and Optimization of 3D-Printed Tablets Containing Mucuna Extracts for Erectile Dysfunction Management: A DoE-Guided Study

Ratchapoom Wattanawiggan [1], Sunee Chansakaow [1], Pensak Jantrawut [1], Pattaraporn Panraksa [1], Jutamas Jiaranaikulwanitch [1], Suruk Udomsom [2], Patnarin Worajittiphon [3] and Pratchaya Tipduangta [1,*]

[1] Department of Pharmaceutical Sciences, Faculty of Pharmacy, Chiang Mai University, Chiang Mai 50200, Thailand
[2] Biomedical Engineering Institute, Chiang Mai University, Chiang Mai 50200, Thailand
[3] Department of Chemistry, Faculty of Science, Chiang Mai University, Chiang Mai 50200, Thailand
* Correspondence: pratchaya.t@cmu.ac.th; Tel.: +66-903-266-555

List of Figures

Figure S1. Images of 13 screening formulation tablets after solidification
Figure S2. Images of 13 screening formulation tablets after printing for calculating shape fidelity
Figure S3. Coded coefficients of factors on (a) viscosity and (b) shape fidelity

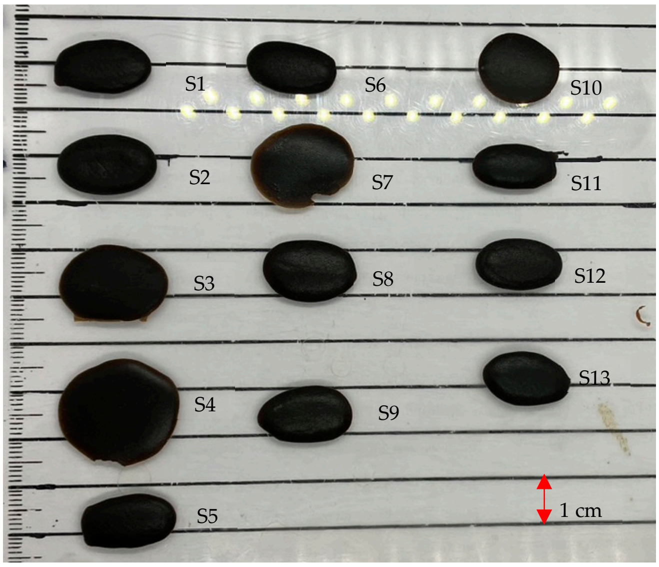

Figure S1. Images of 13 screening formulation tablets after solidification

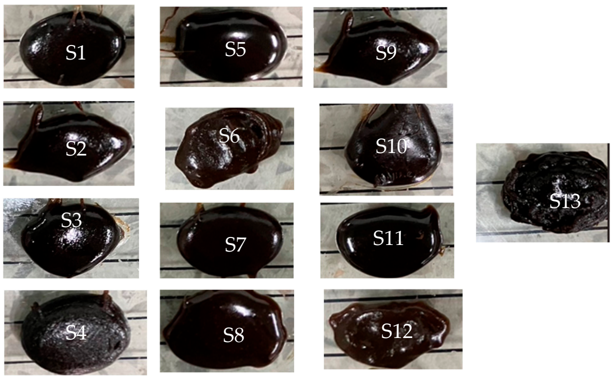

Figure S2. Images of 13 screening formulation tablets after printing for calculating shape fidelity

Coded Coefficients a

Term	Coef	SE Coef	T-Value	P-Value	VIF
Constant	76.37	7.92	9.64	0.002	
PSPC	42.07	9.03	4.66	0.019	1.00
PR	-40.20	9.03	-4.45	0.021	1.00
%IPA	-43.73	9.03	-4.84	0.017	1.00
%glycerin	21.07	9.03	2.33	0.102	1.00
PSPC*PR	-7.8	20.2	-0.39	0.724	4.00
PSPC*%IPA	-12.1	24.4	-0.50	0.653	5.83
PSPC*%glycerin	10.7	16.0	0.67	0.551	2.50
PR*%IPA	43.2	16.0	2.71	0.073	2.50
PR*%glycerin	-21.6	24.4	-0.88	0.442	5.83

Coded Coefficients b

Term	Coef	SE Coef	T-Value	P-Value	VIF
Constant	0.9594	0.0447	21.46	0.000	
PSPC	-0.0902	0.0510	-1.77	0.175	1.00
PR	-0.0236	0.0510	-0.46	0.675	1.00
%IPA	0.0440	0.0510	0.86	0.452	1.00
%glycerin	-0.0439	0.0510	-0.86	0.453	1.00
PSPC*PR	-0.065	0.114	-0.57	0.607	4.00
PSPC*%IPA	0.020	0.138	0.15	0.892	5.83
PSPC*%glycerin	-0.0229	0.0901	-0.25	0.816	2.50
PR*%IPA	-0.0072	0.0901	-0.08	0.941	2.50
PR*%glycerin	0.063	0.138	0.46	0.676	5.83

Figure S3. Coded coefficients of factors on (a) viscosity and (b) shape fidelity

List of Tables

Table S1. Viscosity and shape fidelity of the screening formulations.
Table S2. Optimized formulations design by design of experiment.

Table S1. Viscosity and shape fidelity of the screening formulations.

Formulation (S)	*Mucuna* (g)	PSPC (%)	PR	Glycerin (g)	%IPA (%)	Viscosity (Pas)	Shape fidelity (SF)
S1	2.5	60	0.67:1	0.1	50	160.747±98.562	1.038±0.064
S2	2.5	70	4:1	0.1	50	63.477±1.247	0.745±0.034
S3	2.5	70	0.67:1	0.2	50	330.995±81.940	0.690±0.136
S4	2.5	60	1.5:1	0.15	60	12.745±3.968	1.154±0.057
S5	2.5	50	4:1	0.15	50	65.702±3.288	0.974±0.056
S6	2.5	70	1.5:1	0.1	70	53.233±5.244	0.899±0.11
S7	2.5	50	4:1	0.1	70	14.338±4.615	1.099±0.137
S8	2.5	50	0.67:1	0.1	60	36.643±7.988	1.061±0.066
S9	2.5	70	4:1	0.2	60	80.499±14.054	0.776±0.081
S10	2.5	70	0.67:1	0.15	70	46.770±8.404	1.098±0.057
S11	2.5	50	1.5:1	0.2	50	55.630±1.301	1.073±0.043
S12	2.5	60	4:1	0.2	70	37.049±5.486	0.961±0.081
S13	2.5	50	0.67:1	0.2	70	34.936±10.045	0.903±0.018

Table S2. Optimized formulations design by design of experiment.

Formulations (F)	Mucuna (g)	PSPC (%)	PR	Glycerin (g)	%IPA (%)
F1	2.5	60.0	1.5:1	0.2	70
F2	2.5	70.0	0.67:1	0.2	70
F3	2.5	70.0	1:1	0.2	70
F4	2.5	50.0	4:1	0.2	70
F5	2.5	60.0	1.5:1	0.2	70
F6	2.5	60.0	1.5:1	0.2	70
F7	2.5	60.0	7.57:1	0.2	70
F8	2.5	50.0	0.67:1	0.2	70
F9	2.5	60.0	0.46:1	0.2	70
F10	2.5	45.9	1.5:1	0.2	70
F11	2.5	60.0	1.5:1	0.2	70
F12	2.5	60.0	1.5:1	0.2	70
F13	2.5	74.1	1.5:1	0.2	70
